# Health system barriers to the first dose of measles immunization in Ethiopia: a qualitative study

**DOI:** 10.1186/s12889-024-18132-6

**Published:** 2024-03-01

**Authors:** Meron Addis, Wubegzier Mekonnen, Abiy Seifu Estifanos

**Affiliations:** Department of Reproductive, Family and Population Health, School of Public Health, College of Health Sciences, Addis Ababa University, Addis Ababa, Ethiopia

**Keywords:** Immunization programs, Measles Vaccine, Measles

## Abstract

**Background:**

Ethiopia has made considerable progress toward measles elimination. Despite ongoing efforts, the country remains among those with the highest number of children missing their initial dose of measles vaccine, and the disease continues to be a public health emergency. The barriers within the health system that hinder the first dose of measles immunization have not been thoroughly investigated. This study aims to identify these barriers within the Ethiopian context.

**Methods:**

Qualitative research, using purposive expert sampling to select key informants from health organizations in Addis Ababa, Ethiopia was employed. We conducted in-depth face-to-face interviews using a semi-structured interview guide. A thematic analysis based on the World Health Organization’s health systems building blocks framework was conducted.

**Results:**

The study uncovered substantial health system barriers to the uptake of the first dose of the measles vaccine in Ethiopia. These barriers include; restricted availability of immunization services, vaccine stockouts, shortage of cold chain technologies, data inaccuracy resulting from deliberate data falsification or accidental manipulation of data, as well as data incompleteness.

**Conclusion:**

Our research highlighted significant health system barriers to MCV_1_ immunization, contributing to unmet EPI targets in Ethiopia. Our results suggest that to accelerate the country towards measles elimination, there is an urgent need to improve the health systems components such as service delivery, information systems, as well as access to vaccine and cold chain technologies.

## Introduction

Measles, an infectious disease of significant virulence, can lead to severe complications and death. The risk is particularly pronounced among children under five who have either not been vaccinated or received insufficient vaccination. The World Health Organization (WHO) reported an estimated 128,000 deaths globally attributable to measles in the year 2021 alone [1]. In Ethiopia, there were recorded a total of 182 deaths between 2021 and 2023, resulting in a Case Fatality Ratio (CFR) of 1.1% [2]. This level of mortality is disconcerting, given that measles is a preventable disease with a safe and cost-efficient vaccine.

Measles-containing vaccine (MCV) is the cheapest public health intervention against measles infection in existence. The vaccine has averted 56 million deaths between 2000 and 2021 [1]. However, according to the WHO, the global coverage of the first dose of measles-containing vaccine (MCV_1_) remained between 84% and 86% during 2010–2018 while coverage with MCV_1_ in the WHO African region has stagnated at around 69% since 2013 [3]. In addition, the largest annual increase in children who did not receive MCV_1_ since 2000 was reported in 2020, representing an acute setback in progress toward measles elimination [4].

Ethiopia has expressed commitment to achieving and sustaining regional measles elimination goals set for 2030. The elimination of measles can strengthen immunization programs [5]. The Expanded Program on Immunization (EPI) in Ethiopia, launched in 1980 with six antigens including the measles vaccine, continues to be the current Health Sector Transformation Plan’s (HSTP) core priority [6]. The purpose of the program is to contribute to the reduction of child morbidity and mortality attributable to vaccine-preventable diseases through providing quality immunization services to all eligible children in Ethiopia. The program currently delivers routine measles vaccination services in the country through static, outreach, and mobile health facilities. In addition, with the introduction of new approaches like Reach Every District (RED) and Sustainable Outreach Services (SOS) in 2004 and 2005 respectively, improvement in vaccination coverage has been documented [7, 8].

However, Ethiopia is one of five countries worldwide with the highest number of unimmunized children, per the national immunization coverage estimates of the WHO and United Nations Children’s Fund (UNICEF). Moreover, the country has 1.2 million children who missed out on the first dose of measles vaccine in 2018 [9]. The Mini-Ethiopian Demographic Health Survey (EMDHS) conducted in 2019 has also shown that the percentage of children who have received the first dose of measles immunization in Ethiopia is 59% at the national level. The lowest coverage rate, 28%, was observed in the Afar region [10]. These findings highlight that the elimination goal initially set for 2020 has not been achieved and there is a disparity in MCV_1_ coverage with certain regions exhibiting notably low vaccination rates.

Extensive research has been done focusing on individual-level barriers to measles immunization, and to a lesser extent, on system-level barriers to immunization in general. The individual-level barriers related to socio-demographic characteristics identified in these studies included; young maternal age, high birth order, large family size, rural residence, low economic status, and educational status. awareness and access-related individual-level barriers were; a mother’s lack of knowledge about immunization benefits, negative perception of vaccine side effects, distance to health facility, lack of postnatal follow-up, difficulty tracking defaulters and poor use of maternal health services. The system-level barriers in the Expanded Program on Immunization highlighted by these studies included; unavailability of vaccines on scheduled immunization dates or service interruptions, lack of frequent home visits, poor vaccine storage, poor defaulter tracing, low community engagement, and poor immunization data documentation. These studies predominantly employ quantitative methodologies [11–16]. Nonetheless, there is a paucity of studies focusing on the health system-level barriers specific to the first dose of the measles vaccine. The current study examined barriers to the uptake of the first dose of measles immunization in Ethiopia. The findings from this study will not only augment the existing body of knowledge but also inform policy decisions and more targeted interventions to accelerate the efforts toward the elimination of measles in Ethiopia.

## Methods

### Study design and setting

**Fig. 1:**
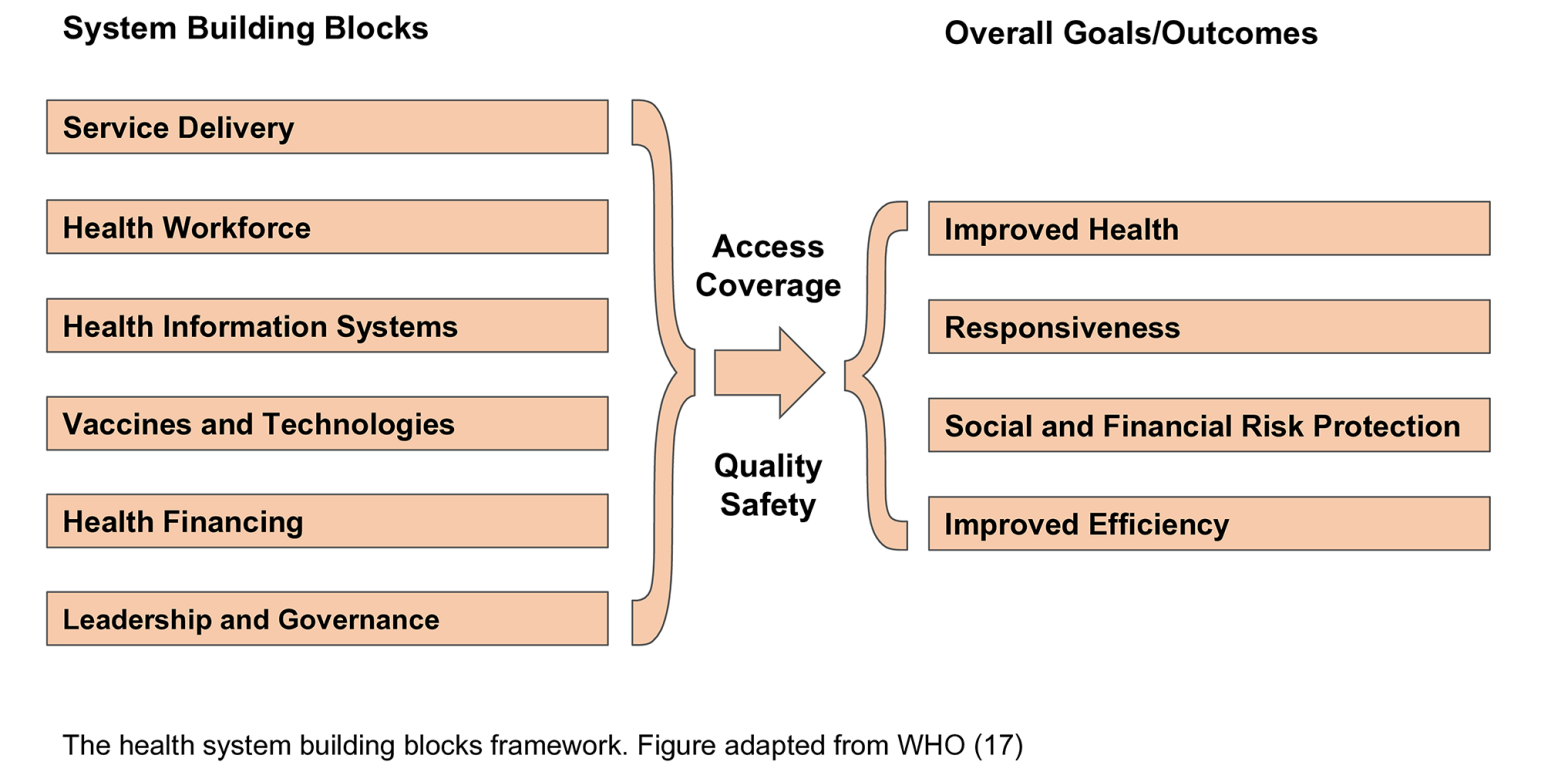
Health systems building blocks framework of the WHO

We conducted a descriptive qualitative case study between October and November of 2022 in Addis Ababa, the capital of Ethiopia. It is widely acknowledged that both governmental and non-governmental organizations (NGOs), situated in Addis Ababa, play a pivotal role in orchestrating the EPI across the nation. Conversely, the responsibilities of the corresponding entities in the other regions primarily encompass overseeing the implementation of measles vaccination and monitoring coverage in their respective regions. In that regard, the city capital was particularly selected to capture the essence of program-related failure to vaccinate the first dose of measles in Ethiopia at a national level. We used the six–building blocks framework developed by the WHO to guide the data analysis for this study [17]. The six core components of the health systems framework are shown in Fig. 1.

The health system building blocks framework. Figure adapted from WHO [17].

### Sampling and study participants

Key informants (KIs), individuals in supervisory and managerial roles with influence and expertise in the immunization program at a national level, were selected through purposive expert sampling with variation in the type of organization they work for. We chose to include KIs from both governmental and non-governmental organizations to obtain a range of experiences and perspectives. Despite the initial plan to interview 30 participants, no new themes and categories were emerging after the 10th interview. Therefore, it was decided that perhaps the data collection had reached a saturation point. A detailed description of the sampling and characteristics of the respondents is presented in Table 1.

The participants were all but one male between the ages of 32 and 51, who have had 5 to 16 years of experience in the EPI program. The respondents were recruited from governmental and non-governmental organizations including; MOH (Ministry of Health), UN agencies (United Nations), implementing partners, and bilateral organizations.

**Table 1:** Demographic characteristics of study participants (*n* = 11)

Characteristics	Frequency
**Sex**	
Male	10
Female	1
**Age**	
30–39 years	8
40–49 years	2
≥ 50 years	1
**Education**	
^1^BSc, ^2^MPH	10
^3^MD, MPH	1
**Job position**	
Technical advisor	5
Program coordinator	4
Immunization officer	2
Program manager	1
**Experience**	
5–9 years	7
10–14 years	3
≥ 15 years	1

Note: ^1^BSc = Bachelor of Science; ^2^MPH = Masters of Public Health; ^3^MD = Medical Doctor

### Data collection procedures

A semi-structured interview guide containing open-ended questions was drafted. The guide included questions to explore barriers to the first dose of measles immunization within the health system, such as: “Could you please point out logistical issues in the implementation of the first dose of measles vaccine schedule, if any?” and we followed up with probing questions such as “could you please elaborate?” to dig deeper. Additionally, to explore the solutions and recommendations related to these barriers, we asked: “What contingencies have the system set in place to remedy these issues?” or “What approaches would you take to alleviate or improve these issues?”. We invited participants for interviews via phone call; only one participant declined an interview due to scheduling conflicts, but none dropped out during the interviews. For those respondents who were available and willing to participate, the principal investigator scheduled interviews at their convenience. Subsequently, the respondents were briefed on the general interview protocol ahead of the schedule, allowing them time to prepare. We conducted eleven in-depth face-to-face interviews in Amharic, the official language of Ethiopia, and took notes of interview contexts, facial expressions, and gestures. On average, the interviews lasted about 35 min; and were held at a space as private as possible, this would generally mean a secluded spot at their place of work or a quiet café. This particular data collection method was chosen due to the interest in obtaining the individual experiences of the respondents.

We obtained informed written consent to be interviewed and audio recorded from all the Participants after the purpose of the study and procedures of how the recorded material would be stored, used, and destroyed were thoroughly explained. Their right to refuse to answer some or all of the questions was allowed and respected. Principles of confidentiality, in agreement with the terms of the ethical approval from Addis Ababa University (AAU), were maintained during data collection. To preserve the anonymity of the respondents, each interviewee was assigned a unique identification number. Furthermore, during the process of coding, any information that could be directly attributed to individual participants was meticulously redacted from the transcripts to uphold the principles of confidentiality and anonymity. The interviews were transcribed and translated verbatim in English by two professional transcribers. The transcribers were given the audio file after each interview was conducted. We reviewed the transcripts and incorporated information on the setting of the interviews and nuances of non-verbal expressions noted during interviews.

### Data analysis

An inductive-deductive thematic analysis was performed in QDA Miner version 3.0. The transcripts were read and re-read thoroughly after each interview with all the relevant excerpts being coded line by line in an iterative approach until a set of initial codes were generated and described. This preliminary list of codes was refined and grouped iteratively until major and/or recurring themes and subthemes were identified with the six components of the health systems building blocks framework of the WHO guiding the structure of the analysis. Finally, patterns of similarities and contradictions in the data were identified and summarized.

## Results

**Fig. 2:**
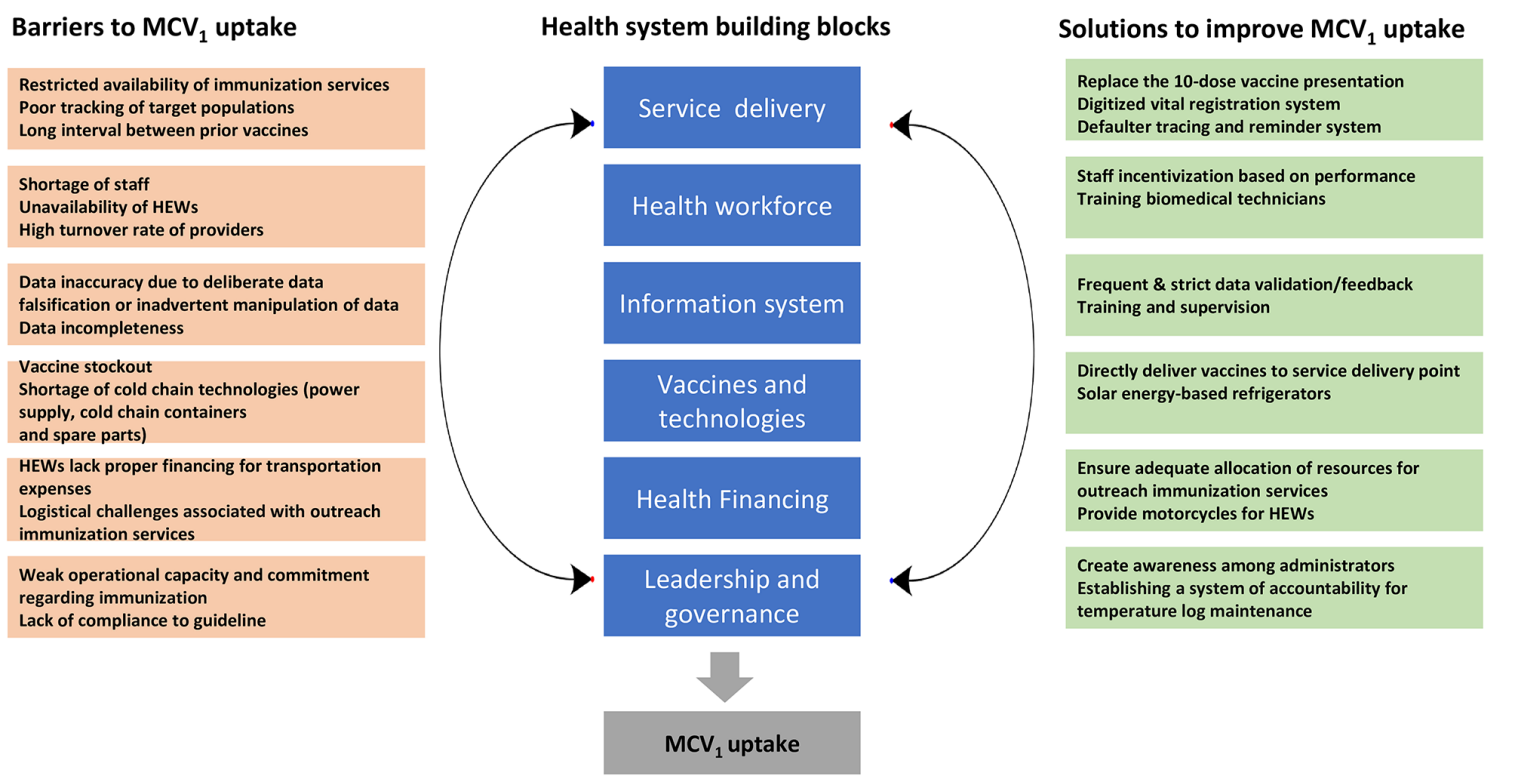
Health systems barriers and solutions to MCV_1_ uptake in Ethiopia

In this section, we present the key themes that emerged from our analysis, all of which fall under the six components of the health system building blocks as shown in Fig. 2. These themes are discussed in depth in conjunction with relevant verbatim quotes extracted from the transcripts.

### Service delivery

Service discontinuity due to restricted availability of measles vaccination service was revealed by the participants as a key barrier in MCV_1_ immunization. According to the participants, measles vaccination services are only available on limited days of the week in most health facilities with intent to minimize wastage. They expanded upon this by saying that the measles vaccine is a 10-dose vial and unused doses are discarded after six hours have elapsed since the vial was first opened, which was well articulated by a program coordinator from an NGO, “one measles vaccine vial is for ten children and providers are often afraid of wasting the vaccine so they tell them [caregivers] to come back on ‘immunization days’, but technically the service is supposed to be given daily right [raises eyebrows]?”. A program manager from an NGO emphasized the restriction of immunization services to curtail wastage, a strategy that holds particular relevance in rural Ethiopia. The rationale behind this approach is the sporadic availability of vaccine recipients during service hours, which often does not justify the opening of a ten-dose vial, “In rural kebeles and Woredas, maybe two or three mothers show up so it is difficult for them [health care providers] to open up a vial intended for 10 children”; he added by saying, “It would be far easier and more economical to use multi-vials”.

Another gap that participants identified is the inability of the immunization system to trace the target population for immunization because vital registration systems are still manual and not comprehensive enough to track the target population for immunization in Ethiopia.

In most other countries, the registration is electronic, but here, it’s manual so the children cannot be easily traced and vaccinated. If it had been electronic, you could see when he [the child] was born, what services he took, and stuff. A proper registration of the vaccinees is crucial. (Technical advisor, governmental organization)

Study participants indicated that the timing of the initial measles vaccine administration could potentially contribute to instances of missed doses. Consequently, the establishment of reminder systems for children due for MCV_1_ immunization is essential.

I believe, it could even decline the [measles] dropout rate that would’ve happened [if it had been given at nine months]; because the mothers often forget to come after several months. The nine-month schedule brings about a wide gap [moving hands], which has always been a problem. (Technical advisor, governmental organization)

Children are vaccinated at the sixth, tenth, and fourteenth weeks [of age], fourteen weeks is like three months and two weeks; so, there is quite some time until nine months. So, the mothers forget it. Unlike prior vaccines that require monthly visits to the health facility, when you tell them to come six months later, they have trouble remembering it; and we don’t have a reminder system in place. So that’s why there are missed doses and the MCV_1_ coverage has always been low, I would say. (Program manager, NGO)

### Health workforce

The current number of healthcare providers and biomedical professionals falls short of the requirements for effective measles immunization. As highlighted by a program coordinator from an NGO “there are not as many health extension workers as needed”. While an immunization officer from a governmental organization underscored the overarching challenge, “Under-one population is very vast, yet the [health] professionals are few”.

In addition, participants discussed the high turnover rate of healthcare providers. Regarding this issue, a technical advisor from a governmental organization commented “There is also some attrition of health professionals. There is even an issue of rotation [of health care providers]”.

It is noteworthy to mention the frequent unavailability of HEWs at their designated workplaces, often attributed to their participation in training sessions, review meetings, or due to maternity leave. As articulated by one interviewee from an NGO, “Health extensions go away for training a lot or review meetings and as you know the health extensions are women and they might be unavailable due to maternity leave; so, there are a lot of holes in the system”.

### Data quality

The participants unanimously agreed that there are inaccuracies and incompleteness within the data related to measles immunization in Ethiopia. “We do see problems with completeness and consistency of the [measles immunization] data a lot of the time” stated an immunization officer from a governmental organization. They also noted, “only in recent years” has the issue garnered “the attention it deserved”.

A program coordinator from an NGO reported, “Data quality issues exist in abundance!”; while a technical adviser from a governmental organization reiterated, “That is a serious issue, to be honest. The numbers represent people; however, some people seem to have lost sight of this basic concept” with a smirk on his face.

Some participants indicated that there has been “some improvement” in immunization data quality, but one participant from a governmental organization expressed concerns that “The data quality problem hasn’t decreased as desired” and suggested “Data validation needs to be done more strictly and more frequently”. Another participant from a governmental organization added that, the inconsistencies in the immunization data are evident by the “huge disparity observed from [data verification] surveys and administrative data” or the “discrepancy seen between results of data validation and reports sent from different facilities”.

Participants explained that the service providers exhibit a propensity to prioritize providing immunization services, often at the expense of ensuring proper registration of the data, this was because they are “overwhelmed with clients” as an immunization officer from a governmental organization described it. A project coordinator from an NGO also recounted that “Most facilities only have one EPI focal but from what I have heard, they give the [immunization] service to 120 or so children on a ‘vaccination day’”.

It was attested by some participants from both governmental and NGOs, that over-reported measles immunization data often come up during data assessments, resulting in overestimated measles vaccination coverages.

Overreporting has become somewhat of a culture here [Ethiopia]. It’s quite baffling really! From the health posts or catchment health centers to the woreda then to the regions, the number of those vaccinated with measles grows and grows [scoffs]. Then we [immunization partners] get a 98% or something [measles vaccination] coverage report, meanwhile you see frequent outbreaks. (Program manager, NGO)

The program manager quoted above hypothesized that overreporting of immunization data is oftentimes due to deliberate manipulation of data, driven by underlying political motivations.

It is completely unethical to report a 90% coverage where children are dying of measles because they were simply not vaccinated. It has become political on its own, they do it because they think; ‘We could lose our leadership position, if the coverage drops’ or ‘If we report a higher coverage, we will be rewarded’.

He also spoke about data falsification during data collection for verification surveys, stating that, “When surveys are done to triangulate those [administrative data] reports, they [data collectors] have been troublesome, they just sit somewhere and fill out the forms”.

On the other hand, the discrepancies in data accuracy could potentially stem from inadvertent alteration of data during the process of recording and summarization. This would happen due to the placement of providers who lack adequate training and experience in managing immunization data. This situation is more prevalent in rural areas, characterized by a high attrition rate among healthcare providers.

Health care providers don’t stay in that position for long; especially in the rural areas because, as you go to places with a [pause] lot of hardships, the probability of health facilities retaining their employees decreases; so then new ones are put in their place. Now these may not have the proper training on registration and tally of immunization data. This leads to inaccurate data. (Program coordinator, NGO)

### Vaccine supply chain

#### Vaccine supply

Although some of the participants reported that there was no significant central vaccine stockout in Ethiopia, the majority of the key informants from both governmental and non-governmental organizations reported stockouts of measles vaccine in health facilities around the county. This particular issue is often driven by delayed vaccine distribution to service delivery points.Although, participants acknowledged that, the efficiency of vaccine distribution has seen considerable improvements in recent years because the vaccines are delivered directly to the service delivery point.

The vaccines [measles containing vaccine] sometimes arrive late [to the health facilities] when distributed, although I would say the distribution has been simplified now; EPSA [Ethiopian Pharmaceutical Supply Agency] directly delivers the vaccines to the Woredas or even [to the] health facilities. (Technical advisor, Governmental organization)

A few of the participants also talked about stockouts of vaccines in health posts due to the dependence of health posts on the catchment health centers for vaccine supplies, as a key barrier in the provision of MCV_1_. The participants explained that HEWs (Health extension workers) are required to procure the vaccines from a catchment health center to deliver outreach services after which they will need to return the empty cold boxes back to the health center; this was considered an inconvenience to HEWs and was related to less frequent immunization sessions.

…They [health extensions] need to go to their catchment health center to bring the vaccines in a [vaccine] carrier and then, they need to return the empty carrier to the health center. The question is, [Tapping the table] is transport or even a proper road service available to them [to fetch the vaccines and return the empty cold box]? No, there is not! Nor are they paid per diem. (Program manager, NGO)

Vaccine stockouts in health facilities may result in the interruption of measles vaccination service, this was demonstrated when participants pointed out the existence of discontinuity of measles immunization service due to stockouts of measles vaccine in health facilities; “There are some [measles vaccine] stock out issues; when mothers show up at a health facility, they are informed to come back” said an immunization officer from a governmental organization. He also mentioned that turning away children due to the unavailability of measles vaccines could result in missed doses, “but not always do we see them back for another try”.

### Vaccine storage and transport

Another concern articulated by a subset of the participants pertains to the scarcity of alternative energy sources, such as solar-powered devices and kerosene to ensure uninterrupted refrigerated storage of measles vaccines. A technical advisor from a governmental organization stated that “SDD [solar derived devices] is provided to health posts and health centers, but it hasn’t been distributed for all [health facilities], thus there are some inconsistencies there”, while another participant from an NGO commented “We can’t say infrastructure is up to the standards everywhere in the country [Ethiopia], power interruption is expected and they [health providers] are required to use kerosene, which isn’t always readily available to them [crossing legs]”, highlighting the necessity of such alternative power supplies given the inevitability of power outage issues in Ethiopia. A program coordinator from an NGO noted that “Solar energy-based fridges are installed in some health facilities, those in remote areas [pause] with electricity issues are given priority” to mitigate the electricity issues.

Beyond power supply issues, the inadequacy of active and passive cold chain containers in the health facilities delivering measles immunization services was raised by participants, a technical advisor from a governmental organization highlighted the issue with an example, “For instance in Oromia region [one of the regions in Ethiopia], only 27% of the health facilities own vaccine carriers; So, you can imagine the potency of the vaccines they are providing, outbreaks are eminent in this situation”.

A few of the participants also mentioned the shortage of spare parts for curative maintenance concerning supply-related barriers in the provision of MCV_1_ immunization; for instance, a participant working in the NGO reported “When maintenance work [cold chain equipment malfunction] is needed, there is usually a shortage of spare parts”.

Furthermore, lack of compliance with guidelines of proper cold chain management by the health care providers was pointed out as an important issue by many of the participants. The participants further elaborated that this included; a lack of temperature monitoring, unattended vaccines in the event of active cold chain failure, or failure to maintain the fridge up to standards. “I have seen uh [pause] in many places where they don’t even record the temperature regularly”, stated a Program coordinator from a non-governmental organization. A similar response was shared by another participant from a governmental organization, “They do not always practice proper maintenance of the fridge and making sure the ice pack is always ready, that has been an issue”.

### Financing

The procurement of measles vaccines in Ethiopia is largely financed by the government and in part by donor partners or NGOs, thus there is no out-of-pocket cost for the vaccinee as confirmed by the participants. One participant from a governmental organization conceptualized this by saying, “…Measles vaccine costs 0.42 USD per dose, 0.20 [USD] is paid by the government and the remaining 0.22 [USD] is covered by the donors”.

A few participants also described how financial constraints affect the delivery of outreach immunization services and supplemental immunization activities (SIAs). ”There are logistics issues [indistinct chatter], especially to implement outreach services and campaigns”, said a technical advisor from an NGO. Another participant from an NGO emphasized that “defaulters are high in measles, thus extra efforts are required to reach the community in measles immunization and this requires more resources”.

Participants also noted that HEWs are required to provide outreach immunization services to the community but are not properly financed to cover their transportation expenses. A program coordinator from an NGO stated that “money for transportation to provide outreach services has been an issue”. A technical advisor from a governmental organization proposed that “HEWs need motorcycles to provide outreach immunization services to the community, and there needs to be a party that can support this”.

### Leadership and Governance

Nearly all the participants from both organizations described the operational capacity of measles immunization in Ethiopia as “huge,” “great,” “very good,” and “good”. Participants from a governmental organization characterized measles as “of a major focus with regards to immunization” thus “there is huge commitment”. Nonetheless, the participants duly noted that the political commitment tends to diminish as it moves down the administrative hierarchy; hence, the overall dedication toward measles immunization is inconsistent.

Measles and polio are of major focus concerning immunization. So, there is a huge political commitment, even when we see it specifically for each vaccine. Although around the lower level, it [political commitment] decreases, which we know of. Attention to immunization is weaker as you go down [the hierarchy of the immunization system]. (Technical adviser, Governmental organization)

Furthermore, there is a lack of awareness among administrators regarding immunization, which hinders their ability to prioritize it effectively.

I think the political commitment to immunization is certainly better in comparison to the other agendas in public health but it is not perfect. You know, [raising eyebrows] administrators are not always consistent in their commitment and some don’t have the proper uh [pause], awareness to care about it enough. (Immunization officer, Governmental organization)

## Discussion

We explored health system-level barriers to immunization of the first dose of measles vaccine in Ethiopia using qualitative data generated with a tool aligned with the six–building block model. A major theme within the service delivery domain that emerged in this study as a barrier to MCV_1_ immunization was the restriction on opening measles vaccine vials. Such restrictions are made with intent to minimize open-vial wastage due to discarding of the remaining vaccine doses that were not administered within six hours of being reconstituted or at the end of an immunization session, whichever occurs first. Vaccinators practice this due to the threshold policy set for them, where they only open a measles vaccine vial if a minimum of six children due for vaccination are present. In such circumstances, a reduced wastage rate is prioritized over vaccine administration at first health facility contact to use scarce resources effectively. Policies for strict vial-opening measures are often a response to specific wastage targets established for immunization programs of Gavi-eligible countries, one of which is Ethiopia [18, 19]. Furthermore, according to a study evaluating the economic impact of different vial-opening thresholds for MCV in using simulation models of the routine immunization supply chains of three African countries, implementing a 30% vial-opening threshold for 10-dose MCVs enhances the overall availability of vaccines across different supply chains and reduce associated medical costs and disability-adjusted life years (DALYs) compared to having no threshold [20].

The revised Multi-Dose Vial Policy (MDVP) statement made by the WHO aims to reduce wastage by allowing opened vials of certain vaccines to be reused up to 28 days after they have been opened under certain conditions. Unfortunately, the MDVP does not apply to lyophilized vaccines without preservatives, including 10-dose measles vaccines [21]. Even though reducing vaccine wastage is an essential goal for immunization programs, it’s also important to ensure that measles vaccines are administered to eligible children. Restrictive vial-opening policy could lead to missed opportunities through delays in vaccination, and by extension lower measles vaccination coverage rates [22]. In addition, there is the risk of vaccines expiring before they can be administered or become damaged while in storage, leading to further wastage. The WHO considers open vial wastage rates of up to 25% for the measles vaccine acceptable [23]. The WHO also considers discarding remaining/unused doses in lyophilized vaccines such as measles 10-dose vials to be an unavoidable reason for wastage [21].

Switching to a lesser vaccine dose presentations could have program benefits such as; vaccine safety, by reducing the risk of cross-contamination from repeated entry by reconstitution syringes and injection syringes for administrations, as well as increased vaccination opportunities and convenience for healthcare providers who would otherwise keep track of the number and volume of doses they withdraw. Conversely, switching to smaller vial sizes from 10-dose could overwhelm the capacities of many storage facilities and transport vehicles as well as increase the cost per vaccinated child. This highlights the need to take into account the cold chain system and resources available when selecting the appropriate vaccine vial [24, 25].Transitioning from 10-dose Measles Containing Vaccine (MCV) vials to 5-dose vials has led to enhanced immunization coverage, reduced vaccine wastage, and increased readiness to initiate a vial. These outcomes can inform approaches aimed at minimizing instances where vaccination is overlooked [26].

The results of our study also suggest that the quality of immunization data in Ethiopia, another key health system component, is poor. Health Management Information System (HMIS) data in Ethiopia has been broadly disparaged for its subpar quality. According to a recent study conducted in Ethiopia, the routine health information systems data quality is below the 80% national expectation, with data completeness, accuracy, and timeliness ranging between 33% and 78% in different parts of the country, Similar results were observed in another study conducted in another part of the country, except for data completeness, which was 86% [27, 28]. A significant discrepancy between the data between the HMIS and the survey data was also shown in a study assessing the quality of maternal and child health data collected through HMIS [29].

Our study demonstrated that inconsistencies with the immunization data could arise anywhere between data collection, recording, or reporting. These could result from inaccurate registration of data by inexperienced and untrained providers, intentional manipulation of data motivated by political considerations as well and falsification of data during verification surveys. Inaccuracy of data whether intentional or unintentional could send the immunization system into a false sense of security and/or obscure decision-making regarding resource allocation, whereas falsifying data from verification surveys defeats the purpose of data quality assessments. Our study identified over reporting as a significant form of immunization data inaccuracy in Ethiopia. As mentioned in our introduction, the coverage for measles in Ethiopia is suboptimal, even lower in certain regions. Our findings suggest a potential over reporting of immunization data in Ethiopia, although primarily in administrative data. This over reporting could cast doubt on the validity of the few measles cases reported in the country, considering the low coverage that might have even be underestimated. A cross-sectional study conducted in Ethiopia also found that the ratio of vaccination data, compared using tallies against the reports, showed evidence of overreporting with 46.5% for the first dose of measles vaccinations [30]. According to our findings, there is evidence to suggest that overreported immunization data could be politically motivated. In a similar report from a scoping review of factors limiting data quality in the expanded programme on immunization in low and middle-income countries (LMIC), incomplete and incorrect data has made it challenging to obtain an accurate overview of immunization coverage, particularly in LMIC; and coverage numerators were seen to be inflated for official reports [31]. This was also revealed by a study conducted in Ghana which found that lower-level health facilities tend to overreport immunization data to avoid being reprimanded by higher-level staff [32].

A recent qualitative investigation conducted in Ethiopia also uncovered that, healthcare providers are subjected to considerable pressure to abstain from reporting authentic data, predominantly from the facility, woreda, or zonal managers. This phenomenon was identified as a systemic driver. Concurrently, at the individual level, there was a reluctance to bear direct accountability or face reproach for poor performance, coupled with an inclination to evade professional complications or potential job termination [33]. It has been suggested by the participants that increasing workers’ capacities through training and supervision, whilst also ensuring adequate and timely feedback to improve data quality issues, the same suggestions were made by the scoping review [31]. Thus, we recommend regular supersvision to enhance these data quality issues that were highlighted through our findings. HMIS is an essential component of health systems, providing data that is used for decision-making and evaluating the performance of healthcare services and programs [34]. Poor data quality has been cited by the Global Vaccine Action Plan (GVAP) as one of the five key priorities that need to be addressed, to realize progress in immunization [35].

Barriers related to access to MCV_1_ vaccines and technologies, a crucial component within the health system, also emerged in this study. The major themes identified in this regard include vaccine stockouts in health facilities due to delayed delivery of vaccines from central hubs or lack of cold chain equipment capacity. Our findings indicate an absence of substantial deficiencies in the supply of measles vaccines at the national level. Nonetheless, our analysis also suggests the occurrence of intermittent disruptions in the availability of vaccines at the points of service delivery. This is in agreement with recent evidence which substantiates that underperforming Immunization Supply Chains have been widely recognized as a significant hurdle to achieving high immunization coverage rates in low- and middle-income countries [36]. Supply chains in some African countries were designed more than 40 years ago, typically adhering to administrative strata, and are now considered outdated and inefficient [37].

Ethiopia has the same experience and has only recently begun the process of redesigning the vaccine supply chain system through the vaccine supply chain transformation initiative that began in 2013, but between 2013 and 2015 the initiative was mainly in the planning stage with little real progress. Even after 2015, progress was slow due to procurement delays. Healthcare services in Ethiopia are delivered by 11 self-administering regions and two city administrations with a Regional Health Bureau (RHB) of their own. The services are further decentralized to Zonal, Woreda/District, and Kebele (smallest administrative unit) levels. Currently, vaccines are being delivered from the center to the regional hub then to the woreda, and in some areas directly to the health facilities, bypassing the zonal level. There are three Hubs, one in each of these regions; Bahir Dar, Mekele, and Jimma. But of the three hubs, Jimma progressed much more slowly due to a lack of involvement and direction from the Oromia RHB, which is located in Addis Ababa, and the local Zonal Health Department (ZHD) may not have the authority to lead the process. Moving forward, the district level is planned to be eliminated, and vaccines will be directly delivered to health facilities. This strategy was also mentioned by the participants of this study. However, there has been little discussion regarding its sustainability or, rather, concerning EPSA’s capacity to manage and sustain a sizable fleet of refrigerated vehicles, particularly for last-mile delivery. [38]. 

Our study also found that health posts rely on catchment health centers for vaccine supply due to a lack of active cold chain equipment capacity, which makes access to immunization challenging. This could be because health posts are based more remotely and are likely to have unreliable electric power supply. The study participants suggested that equipping health posts with solar-derived refrigerators would help address this issue. As part of the vaccine supply chain redesign, 64% of health centers and 25% of health posts in Ethiopia have been equipped with new Solar-based refrigerators as of 2019, and there are plans to equip 100% of health centers and 71% of health posts with these refrigerators over the next few years [38].

The Immunization Agenda 2030 (IA2030) sets an ambitious, overarching global vision and strategy for vaccines and immunization for the decade 2021–2030, and supply chain and logistics are a key area of focus in the first, overarching strategic priority to ensure that immunization programmes are an integral part of primary health care to achieve universal health coverage. One of the cornerstones of an effective national immunization program is for its supply chain to ensure continuous and uninterrupted availability of quality vaccines up to the service delivery point. If vaccine availability is interrupted for any reason, missed opportunities to vaccinate will occur [39]. Addressing missed opportunities for vaccination has become imperative to attain unmet goals in the GVAP [40].

Our study is not without its limitations. The study has a small sample size, we recognize that a larger sample size could have allowed for a more robust analysis. Nonetheless, we are confident that the data we have gathered is valuable and supports our findings. Some participants found it challenging to differentiate their experiences within MCV_1_ from EPI in its entirety. Despite the interviewer’s best efforts to realign participants’ responses to MCV_1_ specifically, some comments may have been more general than expected. In addition, health workforce, financing, and leadership/governance were not frequently discussed or regarded as major sources of issue by the majority of the participants. This could be attributed to the efficient functioning of these three blocks or there are other factors that we didn’t account for in our design. The guiding questions related to these components might not have been adequately detailed or stimulating enough to prompt participants to provide rich data.;ergo future studies could delve deeper into these blocks..

## Conclusion

Our research uncovered health system-level barriers to uptake of the first dose of measles immunization which contribute to the suboptimal MCV_1_ coverage in Ethiopia. The repercussions of suboptimal measles immunization are severe, particularly for the at-risk population of children under five. Our findings identified that the key health systems level barriers lie within the three WHO’s health systems building blocks: service delivery, health information systems, and access to vaccines and technologies. The restricted availability of measles immunization services has contributed to missed opportunities for measles vaccination in Ethiopia. Furthermore, despite the best efforts to streamline vaccine distribution in Ethiopia, access to MCV_1_ remains limited due to frequent stockouts of the measles vaccine in health facilities. Such stockouts can interrupt measles vaccination services, leading to deferred visits that could potentially result in missed doses. Although, it’s essential to ensure timely access to measles immunization to all eligible children to achieve measles elimination.In addition, there were significant concerns regarding data quality in immunization, indicating that Ethiopia’s journey toward improvement in this area is far from over.

## Data Availability

The interview transcripts and coded data supporting the conclusions of this study are not publicly available due to indirectly identifiable individual persons data they contain. However, they are available from the corresponding author upon reasonable request.

## Abbreviations

WHO: World Health Organization
CFR: Case Fatality Rate
MCV: Measles-containing vaccine
MCV_1_: First dose of Measles-containing vaccine
EPI: Expanded Programme on Immunization
HSTP: Health Sector Transformation Plan
RED: Reach Every District
SOS: Sustainable Outreach Services
UNICEF: United Nations Children’s Fund
EMDHS: Ethiopia Mini Demographic and Health Survey
NGOs: Non-Governmental Organizations
KIs: Key Informants
MOH: Ministry Of Health
UN: United Nations
AAU: Addis Ababa University
EPSA: Ethiopian Pharmaceutical Supply Agency
HEWs: Health Extention Workers
SDD: Social Development Department
SIAs: Supplemental Immunization Activities
DALYs: Disability-Adjusted Life Years
MDVP: Multi-Dose Vial PolicyMulti-Dimensional Voice Program
HMIS: Health Management Information System
LMIC: Low- and Middle-Income Country
GVAP: Global Vaccine Action Plan
RHB: Regional Health Bureau
ZHD: Zonal Health Department
IA2030: Immunization Agenda 2030
